# Protocol for the economic evaluation of a community-based intervention to improve growth among children under two in rural India (CARING trial)

**DOI:** 10.1136/bmjopen-2016-012046

**Published:** 2016-11-02

**Authors:** Jolene Skordis-Worrall, Rajesh Sinha, Amit Kumar Ojha, Soumendra Sarangi, Nirmala Nair, Prasanta Tripathy, H S Sachdev, Sanghita Bhattacharyya, Rajkumar Gope, Shibanand Rath, Suchitra Rath, Aradhana Srivastava, Neha Batura, Anni-Maria Pulkki-Brännström, Anthony Costello, Andrew Copas, Naomi Saville, Audrey Prost, Hassan Haghparast-Bidgoli

**Affiliations:** 1 University College London, Institute for Global Health, London, UK; 2 Ekjut, Chakradharpur, Jharkhand, India; 3 Sitaram Bhartia Institute of Science and Research, New Delhi, India; 4 Public Health Foundation of India, New Delhi, India; 5 Department of Public Health and Clinical Medicine, Umeå University, Umeå, Sweden; 6 Department of Infection and Population Health, University College London, London, UK

**Keywords:** Cost-effectiveness, stunting, undernutrition, India, PUBLIC HEALTH

## Abstract

**Introduction:**

Undernutrition affects ∼165 million children globally and contributes up to 45% of all child deaths. India has the highest proportion of global undernutrition-related morbidity and mortality. This protocol describes the planned economic evaluation of a community-based intervention to improve growth in children under 2 years of age in two rural districts of eastern India. The intervention is being evaluated through a cluster-randomised controlled trial (cRCT, the CARING trial).

**Methods and analysis:**

A cost-effectiveness and cost–utility analysis nested within a cRCT will be conducted from a societal perspective, measuring programme, provider, household and societal costs. Programme costs will be collected prospectively from project accounts using a standardised tool. These will be supplemented with time sheets and key informant interviews to inform the allocation of joint costs. Direct and indirect costs incurred by providers will be collected using key informant interviews and time use surveys. Direct and indirect household costs will be collected prospectively, using time use and consumption surveys. Incremental cost-effectiveness ratios (ICERs) will be calculated for the primary outcome measure, that is, cases of stunting prevented, and other outcomes such as cases of wasting prevented, cases of infant mortality averted, life years saved and disability-adjusted life years (DALYs) averted. Sensitivity analyses will be conducted to assess the robustness of results.

**Ethics and dissemination:**

There is a shortage of robust evidence regarding the cost-effectiveness of strategies to improve early child growth. As this economic evaluation is nested within a large scale, cRCT, it will contribute to understanding the fiscal space for investment in early child growth, and the relative (in)efficiency of prioritising resources to this intervention over others to prevent stunting in this and other comparable contexts. The protocol has all necessary ethical approvals and the findings will be disseminated within academia and the wider policy sphere.

**Trial registration number:**

ISRCTN51505201; pre-results.

**Strengths and limitations of this study**

- This protocol will contribute to the limited evidence regarding cost-effective strategies to improve early child growth.
- This replicable protocol can assist in designing economic evaluations for similar complex public health interventions.
- The study design will contribute to our understanding of fiscal space for investments in early child growth.
- The design of the economic evaluation will allow societal, household and provider costs to be disaggregated.
- Community-based interventions may require piloting and modification for use in other settings.

## Background

### Burden of child undernutrition

Undernutrition affects an estimated 165 million children globally and contributes to an estimated 45% of all child deaths worldwide.^1^ Stunting, that is, low length-for-age or height-for-age in young children, is a marker of chronic undernutrition. It is the result of multiple determinants, including long-term consumption of a low-quality diet in combination with morbidity, infectious diseases and environmental risks.^2–4^

Stunting in early childhood is associated with subsequent adverse consequences, including poor cognitive and educational performance, reduced work capacity and lower wages in adulthood.^5^ It also contributes to the intergenerational transmission of poverty.^6^ It is estimated that individual productivity losses attributable to undernutrition are more than 10% of lifetime earnings, and losses to gross domestic product (GDP) may be as high as 2–3% (World Bank, 2006). The burden of undernutrition disproportionately affects the most vulnerable populations in low and middle income countries (LMICs).^7^ ^8^ The WHO has set a global target to reduce stunting by 40% by 2025,^9^ while the 2014 Global Nutrition Report emphasises the importance of nutrition as the foundation on which healthy lives, ‘resilient livelihoods’ and ‘thriving economies’ are built.^10^

Approximately one-third of the world's stunted children live in India. Stunting in India is most prevalent among the poorest households, Scheduled Tribes (ST) and Scheduled Caste (SC) communities.^11^ ^12^ The Government of India has targeted undernutrition through the Integrated Child Development Scheme (ICDS) and the National Rural Health Mission (NRHM). The ICDS distributes supplementary food to children through a network of village-based workers known as ‘Anganwadi’ workers. Launched in 2005, the NRHM (now renamed National Health Mission or NHM) aims to provide accessible, affordable and quality healthcare services to the rural population and vulnerable groups.^13^ Although ICDS services have nationwide coverage, they mainly target children aged 3–6 years, by which time many of the adverse effects of early childhood undernutrition may already be established.

The Indian Government has recently undertaken several reforms in the structure of the ICDS. These include providing supplementary foods to pregnant women, children under 3 years of age and their mothers and improving mothers' feeding and caring practices. The introduction of an additional community-based worker to support these efforts is also being considered. As these reforms are in the earliest stages, evidence on their likely effect is scarce, as is evidence of their cost-effectiveness relative to alternative strategies.^14–16^

### CARING trial

CARING is a cluster-randomised controlled trial (cRCT) located in two districts of Jharkhand and Odisha, eastern India. The trial aims to assess the effectiveness of a complex public health intervention that mirrors the proposed government reforms described above. The intervention involves recruiting and training a new community-based health worker engaged in improving feeding, infection control and caregiving practices for children under 2 years of age through monthly home visits to all children under 2. This new community-based worker also supports monthly women's groups meetings working through a ‘undernutrition-focused’ participatory learning and action cycle. The participatory learning and action cycle involves monthly group meetings for women. The new community-based health worker facilitates the meetings and a problem-solving approach is used to help groups identify community-level health and nutrition problems, and find locally feasible strategies to address the problems identified.

The trial aims to establish whether the intervention is effective and cost-effective in reducing stunting in children under 2, morbidity and mortality, when compared with current practice. In all study areas, we also attempt to strengthen Village Health Sanitation and Nutrition Committees (VHNSCs) as a common benefit to intervention and control clusters.

The CARING trial is described in detail elsewhere.^17^ The purpose of this article is to fully describe the methodology for the economic evaluation of the trial.

### Economic evaluations of community interventions to improve children's growth

Although there is strong evidence demonstrating a significant positive contribution of early childhood nutrition interventions to child survival, growth and development, existing literature provides little data about the cost-effectiveness of such interventions.^15^ The scarcity of empirical cost-effectiveness data for these interventions is particularly acute in LMICs.^15^ ^18^ Policymakers require this information, in addition to evidence on the efficacy and effectiveness of programmes, in order to make investment decisions.

A recent review of the literature on the cost-effectiveness of early child nutrition interventions identified six studies from LMICs.^15^ Two studies examined the cost-effectiveness of breastfeeding interventions in Southern Africa;^19^ a cohort study in KwaZulu-Natal, and Uganda,^20^ and a cRCT in Mbale district, South Africa. Both studies were conducted from the provider perspective. Cost-effectiveness ratios were reported as the cost per mother counselled or cost per visit in Uganda.^20^ In South Africa, the total cost of the implementation was reported, along with the cost per supported month of exclusive breast feeding and the cost per additional or increased month of exclusive breast feeding.^19^ These ratios are not comparable between the two trials and the findings are unlikely to have direct relevance to the Indian context or to the CARING trial, which proposes a more comprehensive nutrition intervention.^17^

The same literature review also identified four trials that have attempted to improve the nutritional status of children more broadly;

- Awasthi *et al*^21^ tested a deworming intervention in a placebo-controlled trial in India.
- Sharieff *et al*^22^ modelled the effects of published home fortification and micronutrient supplementation trials in Pakistan.
- Waters *et al*^23^ trialled a nutrition education programme in a health facility-based cRCT in Peru.
- Wilford *et al*^24^ modelled the community-based management of severe acute malnutrition (SAM) in Malawi.

In the India and Peru studies, cost-effectiveness analyses were conducted from the societal perspective. The India and Peru studies reported incremental cost-effectiveness ratios (ICERs) per case of stunting averted of $34.67^i^ and $55.16, respectively. The Peru study reported the cost per death averted at $1952. In Pakistan and Malawi, cost-effectiveness analyses were conducted from the provider perspective and as such their findings cannot be compared directly with the other two studies. They did, however, report a cost per disability-adjusted life year (DALY) averted of $12.20 and $42, respectively, which can then be compared with other ‘DALY averting’ interventions. In addition, the Pakistan study reported a cost per death averted of $406.

The proposed economic valuation of the CARING trial will be the first to assess the cost-effectiveness of combining participatory women's groups with home visits to reduce stunting. This economic evaluation will also employ a societal perspective, thus adding significantly to our understanding of the cost-effectiveness of this intervention and the likely fiscal space needed for its wider implementation. ICERs will be reported for cases of stunting averted, deaths averted and DALYs averted, in order to maximise comparability with other trials and ensure the usefulness of the economic evaluation as a resource allocation tool.

### Aim and objectives

The aim of the CARING economic evaluation is to measure the cost-effectiveness of a community intervention that includes participatory learning and action with women's groups and home visits to improve the growth of children under 2. Activities to strengthen local VHSNCs will be undertaken in intervention and control arms.

The specific objectives of the economic evaluation are:

1. To measure the costs of setting up and implementing the community intervention to improve children's growth and the strengthening of VHSNCs.
2. To estimate the costs to the health system, of (a) treatment for SAM provided at Malnutrition Treatment Centres (MTCs) or Nutritional Rehabilitation Centres (NRCs); (b) increased care seeking at Primary Health Centres for childhood illnesses, as a result of the programme.
3. To measure the direct and indirect cost of the intervention to the health system, when existing community health workers may take on a greater workload resulting from the intervention.
4. To measure the direct and indirect cost of the participation of community leaders or VHSNC members in participatory meetings or other activities related to the intervention.
5. To calculate the household costs of care seeking related to undernutrition and any costs of changing feeding and caring behaviours, for example, the possible costs of buying additional/different foods.
6. To estimate the incremental cost-effectiveness of the intervention combined with strengthening of VHSNCs, as compared with strengthening VHSNCs alone, where all new activities are delivered in addition to existing government programmes.

## Methods

### Study design

A cost-effectiveness analysis of the CARING trial, a cluster-randomised trial that compares a community intervention to improve children's growth combined with the strengthening of VHSNCs, with strengthening VHSNCs only.^17^ We will estimate the total and incremental costs of the intervention prospectively from a societal perspective, measuring programme, provider and household costs.

### Study setting

As described fully in the trial protocol,^17^ the CARING trial takes place in West Singhbhum (Jharkhand state) and Kendujhar (Odisha state) districts in India. Odisha and Jharkhand are among the poorest states in India, with high rates of child mortality and undernutrition,^25^ ^26^ and female literacy rates below 50%.^27^ In 2010, under-5 mortality was estimated 61 and 83 per 1000 live births in rural Jharkhand and Odisha, respectively.^28^ ^29^ In Jharkhand over 50% of children are stunted, and in Odisha over 45% are stunted.^25^ ^26^ The study districts are predominantly rural, with a combined total population of ∼3.3 million.^17^

Community-based health and nutrition services for mothers and children in the study villages are provided by three community workers: an Anganwadi worker (AWW), an Accredited Social Health Activist (ASHA) and an Auxiliary Nurse Midwife (ANM). AWWs provide supplementary nutrition to pregnant women, breastfeeding mothers and children aged 6 months to 6 years. ASHAs support community members in accessing essential healthcare services and promote institutional deliveries. ANMs provide services such as childhood immunisation and essential medicines. Community-based services are supplemented with care from primary care centres, MTCs that specialise in the treatment of SAM and hospitals.

### Study population

The CARING trial area includes 120 geographic clusters of around 1000 population, typically a village and any nearby hamlets. These clusters were purposively selected based on their population size. The study participants are pregnant women identified in the third trimester of pregnancy and all children born to pregnant women recruited between 1 October 2013 and 10 February 2015. Mothers and their children are followed up for a period of 18 months after the birth of the child.

### Trial design

In the CARING trial, a total of 120 purposively selected geographical clusters, with an estimated total population of 121 531, were randomised to the two trial arms. The 60 clusters in the intervention arm received home visits, participatory learning and action meetings and support to VHNSCs as described further below. The 60 clusters in the control arm received only the support to VHNSCs. Study participants are pregnant women identified in the third trimester of pregnancy and their children (n=2520). The randomisation, which took place in July 2013, was stratified by district and by number of hamlets per cluster. The trial is ongoing and is due to finish in 2016.

In the intervention clusters, a community intervention to improve children's growth involving home visits to all children under 2 and monthly participatory learning and action meetings with women's groups, combined with VHSNC strengthening activities, is being implemented. In the control clusters, only VHSNC strengthening is implemented. In the following section, we present a brief description of the intervention strategies. A full description of the intervention design within the CARING trial is described in detail elsewhere.^17^

### Community intervention to improve children's growth

The community intervention involves a community-based worker (known as Suposhan Karyakarta or SPK) carrying out two main activities: (1) undertaking monthly home visits and (2) facilitating monthly women's group meetings. An SPK will visit all pregnant women at least once during their pregnancy, and all mothers of children under 2 on a monthly basis, to counsel mothers on appropriate feeding, infection control and care giving. She also facilitates a cycle of monthly women's groups, where the discussions will follow a participatory learning and action cycle focused on maternal health, child health and nutrition. A four phase ‘participatory learning and action cycle’, adapted from previous studies,^30^ will guide the groups to identify and prioritise maternal and child health and nutrition problems, decide on locally appropriate solutions, then implement and evaluate the solutions. These meetings primarily target pregnant and lactating mothers, mothers of children under 2 and adolescent girls. In home visits and women's group meetings, the SPK uses a problem-solving approach.

### Strengthening Village Health Sanitation and Nutrition Committees (VHSNCs)

In intervention and control areas, activities are undertaken to strengthen the capacity of VHSNCs, which are village-level government-mandated bodies. VHSNCs are responsible for assessing the health needs of their community, preparing and implementing village health plans and monitoring the delivery of local health and nutrition services. During the study, several participatory meetings will be held with VHSNCs. These meetings will primarily aim to sensitise VHSNCs on issues related to inequity and exclusion in service provision, in addition to identifying and addressing gaps in health and nutrition services within their community.

### Measurement of health outcomes/effectiveness

#### Primary outcome

The primary outcome of the CARING trial is children's length-for-age z-scores.^17^ The trial was powered to detect a 0.15 SD difference in LAZ between intervention and control at the 0.05 significance level. A greater sample size would be required to detect a realistic change in the prevalence of stunting: our sample size allows us to detect a 13% reduction in stunting prevalence, which is an unfeasible effect for a 24 months intervention without food supplementation. Cost-effectiveness analyses focusing on cases of stunting averted would be useful to inform policymakers' decisions. We therefore propose to conduct a cost-effectiveness analysis for cases of stunting averted if there is evidence of impact on the prevalence of stunting in children at 18 months at the 0.1 significance level or below. Using these z-scores, we will calculate the prevalence of stunting among children at 18 months and calculate the number of cases of stunting averted. Cases averted will be calculated as the difference between the expected and the actual number of cases using the adjusted OR.

#### Secondary outcomes

The trial has a number of secondary outcomes described in detail in the trial protocol.^17^ Cost-effectiveness analyses will be conducted for any reduction in three key secondary outcomes—underweight, wasting and infant mortality—where a significant impact on these outcomes is observed at the 0.1 level.

ICERs will thus be calculated for wasting, underweight and infant mortality, if a significant effect is observed at a p value of 0.1 or less. Estimates of life years saved will be calculated for infant mortality averted by the intervention if a significant finding is observed. Although no agreed DALY weight currently exists for undernutrition in any form, expected ‘DALYs averted’ will be modelled using mortality averted by the intervention if significant, and evidence from the wider literature about morbidity gains from averting stunting, wasting and underweight in young children.^31^

### Equity impact

As the gains from such a complex intervention may not be equitably shared among the target population, the equity impact of the intervention will be analysed within the economic evaluation. Primary and secondary outcomes will be decomposed according to the socioeconomic status of ‘recipient households’ and the economic evaluation stratified accordingly. To account for the fact that many households in these districts will be asset or cash poor, a multidimensional poverty score will be used to define households' socioeconomic status.^32–34^ These stratified, subgroup analyses may enable us to further verify the argument by Carrera *et al*,^35^ that investing in the most deprived communities is more cost-effective than the same investment in less deprived communities.

#### Identification, measurement and valuation of resource use

These analyses will measure the cost-effectiveness of the intervention from a societal perspective. This perspective is the most comprehensive, taking into account costs incurred by the programme, implementing agencies or donors, providers and households.^36^ This perspective determines the time horizon of any modelling, the choice of costs and outcomes to be included in the analysis, and the measurement and valuation of costs and consequences of the intervention.^37^ ^38^ In this section, we will describe in detail the proposed methods for measuring and valuing programme costs, provider costs and household costs. Programme costs include those incurred by the implementing agencies, that is, University College London, the Public Health Foundation of India (PHFI) and Ekjut (http://www.ekjutindia.org), a civil society organisation leading the study. The provider or health system costs are those incurred by MTCs, Primary Health Centres, community workers and VHSNCs. The household or user costs include those incurred by mothers and their families. Table 1 shows the flow of cost data collection described further below.

**Table 1:** Steps in cost data collection

Cost category or perspective	Type of costs	Description	Source	Sample size
Project/programme	Direct	Costs of implementing the intervention	Project accounts of implementing agencies	N/A
Provider/health system	Direct	Costs of referrals made to MTCs	Project records for number of referrals, MTC costing study for unit costs of services	All referrals made in intervention and control clusters
	Indirect	Opportunity cost of increase in workload of CHWs	Time use survey with CHWs	A purposive sample of CHWs in both arms will be selected for time use interviews
		Opportunity cost of time spent by VHSNC members in meetings	Project records	All meetings held and number of people attending the meetings will be recorded
Patients/households	Direct	Household expenditure on food	Household consumption survey	A random subsample of 300 households
		Cost of care seeking for mothers and children	3-month and 18-month follow-up surveys with mothers	All participants in the study
	Indirect	Opportunity cost of participation in groups and home visits	Household time use survey	A random subsample of 120 mothers, with children aged 13–18 months
		Opportunity cost of changing health, nutrition or stimulation behaviour	Household time use survey	A random subsample of 120 mothers, with children aged 13–18 months

MTC, Malnutrition Treatment Centres; VHSNC, Village Health Sanitation and Nutrition Committee.

#### Programme-related costs

We use a step-down costing methodology^39^ whereby costs from programme accounts are entered into a customised tool created in MS Excel. Cost data are entered regularly into the tool, which is adapted each year to reflect the changing cost structure of the trial at different phases of activity. Using a step-down method, the main worksheets for entering data allocate costs to one of the following categories: staff, material, capital and joint costs. Costs are also divided into start-up and implementation costs, and between intervention, monitoring and evaluation and research costs. As these data are financial or accounting costs, they are converted to economic costs. This means that capital costs are annualised over their expected useful life, and any donated goods or volunteer time appearing as a zero costs in accounting data—or not appearing in the accounting data in any form—will be added to the cost sheets and assigned their current market value.^40–42^ Key informant interviews with project leads assist in identifying any donated goods requiring revaluation and in allocating joint costs between programme components. The allocation of joint costs to programme components and activities is also informed by monthly staff time sheets.

Summary Excel worksheets present the costs by programme component such as women's groups or health service strengthening. A single summary worksheet also summarises the total cost data, allows effect data to be entered and calculates the cost-effectiveness results.

### Provider or health system costs

Provider costs or costs to the health system will be calculated for MTCs, Primary Health Centres, community workers and VHSNCs.

#### Malnutrition treatment centres and primary health centres

MTCs provide care for SAM. Demand for these services may increase if SAM is better identified in the community and/or systems for referring children to MTCs are improved. Conversely, demand for these services may decrease if SAM is prevented by improved early child nutrition.

We will estimate any change in the demand for MTC services resulting from the intervention, and the concomitant value of any additional care provided. The trial's routine monitoring forms will capture the number of children admitted for SAM at MTCs. Differences in admissions between intervention and control areas will be attributed to the trial activities. Primary data on the average unit cost of care for SAM will be collected from the two MTCs in the project area. A simple cost-capture form was developed for facility data collection after an introductory meeting with centre managers. Data from the cost-capture form will be used to complement existing data from centre reports, patients’ records and published national and state reports relating to MTCs. Costs of services provided by the MTCs will be calculated using a step-down approach.^39^

For Primary Health Centres, any change in demand for primary care will be identified in the trial's routine monitoring forms as described above. Any cost of that change in demand will be calculated using published data on the unit costs of treatment at Primary Health Centres.^43^

#### Community workers

The CARING intervention may increase the workload of community health workers by increasing demand for their services. Conversely, the new community-based worker (or SPK) introduced by the CARING trial, together with a stronger emphasis on preventive activities, may decrease the workload of existing community workers.

Changes in the number of referrals, demand for counselling, uptake of immunisation and presentation for growth monitoring services will be measured with routine trial monitoring forms. The value of the change in demand for these services will be calculated using process data on the average time spent on each activity, and publically available data on the salaries paid to community workers.

#### VHSNCs

During the course of the intervention, several participatory meetings will be held with VHSNC members. The number of meetings, their duration and participation is being documented by the project. The opportunity cost of the time spent by the VHSNC members will be measured as a proportion of their salary where they are paid a salary—or as a salary equivalent, where they are working as volunteers.

### Household or user costs

CARING may influence household costs in a number of ways. Changes in the quantity, variety or quality of food fed to pregnant women and young children, may increase household expenditure on food. The intervention may also increase care seeking. The effect on health spending of this change may be positive or negative. Increases in care seeking from a very low base may increase total spending on care. Conversely, a shift from curative to preventive care seeking may decrease spending on care. Similarly, improved early child nutrition that averts severe malnutrition and related illnesses, may also reduce the need for care seeking. Finally, as the intervention seeks to change health and nutrition-related activities, it may also change the allocation of time to these activities. Participation in women's group meetings and in home visits will also have a direct time ‘cost’.

Changes in food expenditure are measured with a comprehensive household consumption and expenditure survey conducted on a random subsample of 300 households (150 per trial arm). The consumption and expenditure model is conducted twice for each participating household, with the two data collection points 1 year apart. This allows the measure of interest to be the change in expenditure over a 1-year period, with that change compared between intervention and control areas.

Changes in health spending are measured with a set of questions on care seeking collected from all the mothers recruited in the project. Care seeking data are also collected at two time points in the trial; first when the pregnant mother is recruited and again when the baby is 18-month old. In this instance, the measure of interest is not the change in spending over that time period, but a comparison of absolute levels of spending between intervention and control families with children of the same age.

Finally, the time cost of group participation, participating in the actions taken by the group, engaging with home visits or changing health, nutrition or stimulation behaviours will be measured using a time use survey to be conducted on a subsample of 120 women, equally distributed across the intervention and control arms.

### Cost-effectiveness analysis

The cost-effectiveness analysis will be conducted as a within-trial analysis using the intention-to-treat results, and will be presented in terms of ICERs, calculated as the arithmetic mean difference in cost between the community intervention combined with VHNSC strengthening versus VHNSC strengthening only, divided by the arithmetic mean difference in effect.^37^ ^44^

ICERs will be calculated for the primary outcome measure, that is, cases of stunting prevented, and for selected secondary outcomes and summary measures, including cases of wasting and underweight prevented, cases of infant mortality averted, life years saved and DALYs averted.

A number of sensitivity analyses will be carried out in order to assess the impact on the cost-effectiveness results, of changes in variables and parameters with the greatest uncertainty or with the greatest impact on the total costs. Cost-effectiveness acceptability curves will be generated to further describe uncertainty around the cost estimates.^45^

Costs will be presented in 2016 prices in Indian rupees and International Dollars (INT$). All costs will be adjusted for inflation using the Indian Consumer Price Index (CPI) and will be converted to 2016 INT$ using the 2016 Purchasing Power Parity (PPP) conversion factor for India. Moreover, costs and outcomes will be converted to present values using an annual discount rate of 3% in the base case, and annual rates of 0% and 6% in sensitivity analysis.

The affordability of the intervention will be explored using a range of criteria, including the WHO-CHOICE criteria,^46^ together with an analysis of fiscal space for programme delivery using a generalised fiscal space assessment method^47^ ^48^ and probabilistic analyses to determine a set of cost-effectiveness thresholds.^45^ ^49^ These analyses will also enable the exploration of a multicriteria decision analysis framework for resource allocation to this and other similar interventions to reduce stunting and wasting in young children.^50^ A wide range of affordability measures have been selected, in part, due to the paucity of evidence on the cost-effectiveness of comparable interventions. This precludes the use of simpler league tables and requires a wider range of affordability measures against which subsequent analysts may compare their own findings.

### Conclusion

This paper constitutes the first published protocol for the economic evaluation of a complex public health intervention to improve child growth. Trial groups are encouraged to publish protocols in order to improve the rigour of any subsequent impact assessment. If economists were encouraged to publish the protocols for complex economic evaluations in the same way, we may see a similar rise in the rigour of the conduct of these studies, along with greater comparability between findings.

The proposed analyses aim to assess the cost-effectiveness of a community intervention to improve children's growth. The protocol, which will adhere to internationally recognised guidelines for conducting and reporting economic evaluation studies,^37^ serves to heighten the transparency of the economic evaluation and planned analyses. The findings from this study will inform policymakers about the relative value for money of this intervention and the likely fiscal space required to scale up the intervention in three stages; (i) to all rural populations in Jharkhand and Odisha, (ii) to other states with a high burden of undernutrition and (iii) national scale up. This evidence will contribute significantly to the scarce evidence regarding the cost-effectiveness of community interventions in reducing child undernutrition.

## Footnotes

i These costs are reported in International Dollars, at 2005 prices. Although they were not reported in this currency within the original publications, they were converted to a common currency and base year by Batura *et al*^40^ to facilitate comparison where possible.
